# The role of the C8 proton of ATP in the catalysis of shikimate kinase and adenylate kinase

**DOI:** 10.1186/1471-2091-13-15

**Published:** 2012-08-10

**Authors:** Colin P Kenyon, Robyn L Roth

**Affiliations:** 1 CSIR, Biosciences, Meiring Naude Road, Pretoria, 0001, Gauteng, South Africa

## Abstract

**Background:**

It has been demonstrated that the adenyl moiety of ATP plays a direct role in the regulation of ATP binding and/or phosphoryl transfer within a range of kinase and synthetase enzymes. The role of the C8-H of ATP in the binding and/or phosphoryl transfer on the enzyme activity of a number of kinase and synthetase enzymes has been elucidated. The intrinsic catalysis rate mediated by each kinase enzyme is complex, yielding apparent *K*_M_ values ranging from less than 0.4 μM to more than 1 mM for ATP in the various kinases. Using a combination of ATP deuterated at the C8 position (C8D-ATP) as a molecular probe with site directed mutagenesis (SDM) of conserved amino acid residues in shikimate kinase and adenylate kinase active sites, we have elucidated a mechanism by which the ATP C8-H is induced to be labile in the broader kinase family. We have demonstrated the direct role of the C8-H in the rate of ATP consumption, and the direct role played by conserved Thr residues interacting with the C8-H. The mechanism by which the vast range in *K*_M_ might be achieved is also suggested by these findings.

**Results:**

We have demonstrated the mechanism by which the enzyme activities of Group 2 kinases, shikimate kinase (SK) and adenylate kinase 1 (AK1), are controlled by the C8-H of ATP. Mutations of the conserved threonine residues associated with the labile C8-H cause the enzymes to lose their saturation kinetics over the concentration range tested. The relationship between the role C8-H of ATP in the reaction mechanism and the ATP concentration as they influence the saturation kinetics of the enzyme activity is also shown. The SDM clearly identified the amino acid residues involved in both the catalysis and regulation of phosphoryl transfer in SK and AK1 as mediated by C8H-ATP.

**Conclusions:**

The data outlined serves to demonstrate the “push” mechanism associated with the control of the saturation kinetics of Group 2 kinases mediated by ATP C8-H. It is therefore conceivable that kinase enzymes achieve the observed 2,500-fold variation in *K*_M_ through a combination of the various conserved “push” and “pull” mechanisms associated with the release of C8-H, the proton transfer cascades unique to the class of kinase in question and the resultant/concomitant creation of a pentavalent species from the γ-phosphate group of ATP. Also demonstrated is the interplay between the role of the C8-H of ATP and the ATP concentration in the observed enzyme activity. The lability of the C8-H mediated by active site residues co-ordinated to the purine ring of ATP therefore plays a significant role in explaining the broad *K*_M_ range associated with kinase steady state enzyme activities.

## Background

It has been demonstrated that the adenyl group of ATP plays a direct role in the control of ATP binding and/or phosphoryl transfer within a range of kinase and synthetase enzymes [1,2]. The role of the ATP C8-H in the binding and/or phosphoryl transfer activity of a number of kinase and synthetase enzymes was elucidated in comparative enzyme activity assays using ATP and ATP deuterated at the C8 position. These comparisons demonstrated that a primary kinetic isotope effect (KIE) was involved in all cases.

Historically, the kinases have been classified into 25 families of homologous proteins, with the families assembled into 12 fold-groups based on the similarity of their structural folds [3,4]. This classification relays little information on the catalytic mechanisms employed in nucleotide binding and phosphoryl transfer. The functionality required for the catalysis and regulation of phosphoryl transfer was found to be conserved within the families or fold-groups. As a result, a number of conserved mechanisms were proposed, wherein the C8-H of the adenyl moiety was found to play a direct role in the control of phosphoryl transfer [2]. These mechanisms were developed using structurally conserved amino acid residues within hydrogen-bonding distance of a nucleotide in the active sites of crystallised kinases of a particular mechanistic class. On the basis of these conserved mechanisms, the role of the nucleotide C8-H in initiating the formation of a pentavalent phosphorus intermediate from the γ-phosphate of ATP and the substrate nucleophile was defined. All the kinase mechanistic classes were clustered into two proposed mechanisms depending on how the C8-H is induced to be labile, namely by either the co-ordination of a backbone carbonyl to C6-NH_2_ of the adenyl moiety (a “push” mechanism), or based on the protonation of N7 of the adenyl moiety (a “pull” mechanism). Associated with both the “push” and “pull” mechanisms is a proton transfer cascade via the tri-phosphate backbone, initiated from C8-H, and mediated by specific conserved amino acid residues unique to a particular mechanistic “class” of kinases, that culminates in a pentavalent species formed between the γ-phosphate of ATP and the substrate nucleophile. The “push” mechanism was defined within the Group 2 kinases [2]. The Group 2 kinases are based on the kinase organization outlined by Cheek *et al.*[3]).

Two examples of enzymes falling within the Group 2 kinases are *Mycobacterium tuberculosis* shikimate kinase (SK) and human adenylate kinase 1 (AK1). SK belongs to the nucleoside monophosphate (NMP) kinase structural family, a sub-family of the P-loop containing nucleoside tri-phosphate hydrolase superfamily (Pfam Clan: *AAA*:CL0023) [5]. The shikimate pathway is a seven-step biosynthetic route that links the metabolism of carbohydrates to the synthesis of aromatic amino acids by the conversion of erythrose-4-phosphate to chorismic acid [6]. SK (EC 2.7.1.71), the fifth enzyme in the shikimate biosynthetic pathway, catalyzes phosphate transfer from ATP to the 3-hydroxy group of shikimate, forming shikimate-3-phosphate. Adenylate kinases (AKs) contribute to the homeostasis of adenine nucleotides by maintaining intracellular nucleotide pools. Six isoenzymes of adenylate kinase have been identified in mammalian cells with different subcellular localization and substrate specificity [7,8]. The AKs (ATP:AMP phosphotransferases, EC 2.7.4.3) catalyse the reversible transfer of the γ-phosphate group from a phosphate donor (ATP, GTP, CTP, ITP) to ADP, with the phosphate donor usually ATP. AK1 also belongs to the P-loop containing nucleoside tri-phosphate hydrolase superfamily (Pfam Clan: *AAA*:CL0023). There is a size variation among the isoenzymes: AK1, AK5 and AK6 are short type AKs, while AK2, AK3 and AK4 are long type AKs that contain a 27 amino acid insertion sequence in the central portion of the protein [8]. The mammalian AKs have a distinct intracellular compartmentalization, with AK1 in the cytosol, AK2 in the intermembrane space of mitochondria, AK3 in the mitochondrial matrix, AK4 being mitochondrial in nature, AK5 (unknown) and AK6 in the nucleus [9-15].

**Figure 1:**
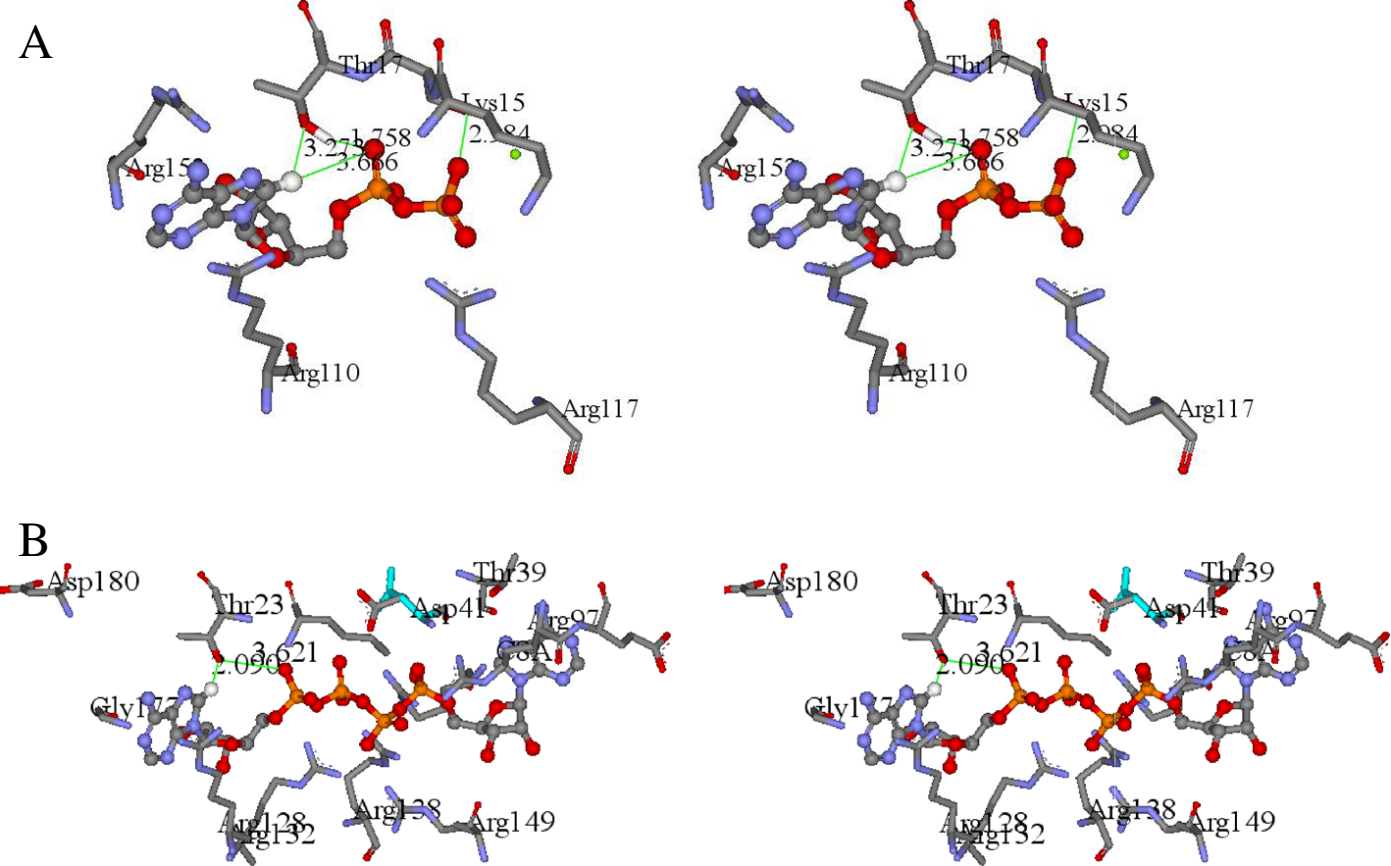
**Push mechanism” catalytic amino acid residues.** Amino acid residues making up the “push” mechanism within the active sites of SK (**A**) and AK1 (**B**). Shown are the protein backbone carbonyl associated with the C6-NH_2_, the Thr associated with the proton transfer from C8H to the α-PO_4_ (SK, Thr17; AK1, Thr 23), the Arg associated with C8 protonation (SK, Arg110: AK1; Arg128), the Arg coordinated to the α-PO_4_ and β-PO_4_ (SK, Arg117; AK1, Arg132), Lys associated with the γ-PO_4_ protonation (SK, Lys15; AK1; Lys21). The adenylate kinases have two nucleotide binding sites and the equivalent amino acid residues in the second AK1 binding site are carbonyl associated with the C6-NH_2_, the Thr associated with the proton transfer from C8H to the α-PO_4_ (Thr 39), the Arg associated with C8 protonation (Arg97), the Arg coordinated to the α-PO_4_ and β-PO_4_ (Arg44/138), Lys associated with the γ-PO_4_ protonation (Lys21).

Comparison of the active sites of SK and AK1 clearly indicates structural homology of the key amino acids making up the “push” mechanism (Figure 1) [2]. Shown are the protein backbone carbonyl associated with the C6-NH_2_, the Thr associated with the proton transfer from C8-H to the α-PO_4_ (SK, Thr17; AK1, Thr23), the Arg associated with C8 protonation (SK, Arg110: AK1; Arg128), the Arg coordinated to the α-PO_4_ and β-PO_4_ (SK, Arg117; AK1, Arg132), and the Lys associated with the γ-PO_4_ protonation (SK, Lys15; AK1; Lys21). The AKs have two nucleotide binding sites and the equivalent amino acid residues in the second AK1 binding site are Thr 39 (proton transfer from C8H to the α-PO_4_), Arg97 (C8 protonation), Arg44/138 (coordinated to the α-PO_4_ and β-PO_4_), and Lys21 (γ-PO_4_ protonation). A site-directed mutagenesis (SDM) programme was initiated to ascertain the necessity of these identified residues for catalysis, as well as to determine the functionality of the mutated enzymes. The KIE resulting from the deuteration of ATP at the C8 position was also determined for each enzyme. Kinetic isotope effects (KIE) are broadly classified into primary, secondary and steric effects. The extent of proton/deuterium KIEs is estimated from the rate constants (KIE = *v*_H_/*v*_D_) and a KIE ≥ 2 is strong evidence that the bond to the isotopically substituted hydrogen atom is being broken in the rate determining step of the reaction for the primary KIE [16,17]. The calculated maximum for the KIE involving C-H bonds is approximately 7 at room temperature as determined by the difference in the zero-point energy-difference between the bond to the deuterium and the bond to the hydrogen, at least in isolated chemical systems. The secondary deuterium KIEs are defined as the isotope effect when the bond to the isotopically substituted atom is not cleaved but occur as a result of a hybridization change, with the KIE ranging between 0.7 and 1.4. Steric effects influence KIEs to the same extent as the mechanistic events affording secondary KIEs. The observed primary KIEs resulting from ATP C8-H/D substitution in a variety of kinases, together with the proposed mechanisms associated with each kinase family, prompted the undertaking of this SDM programme. The aim was to determine the specific mechanistic role of the conserved amino acid residues in the active site, required both in binding of ATP as well as the initiation phosphoryl transfer in SK and AK1 [1,2]. The SDM and the associated steady-state kinetics for each mutated enzyme in the presence of ATP and C8-D ATP was used to delineate the “push” mechanism, as defined by Kenyon *et al.* (2012). This was done by comparing the enzyme activity in the presence of ATP with that using C8D–ATP as a measure of the intrinsic KIE for that enzyme, in conjunction with SDM of the conserved amino acids implicated in the “push” mechanism as a probe of the mechanistic role of these residues.

## Results

### SDM of SK and AK1

**Figure 2:**
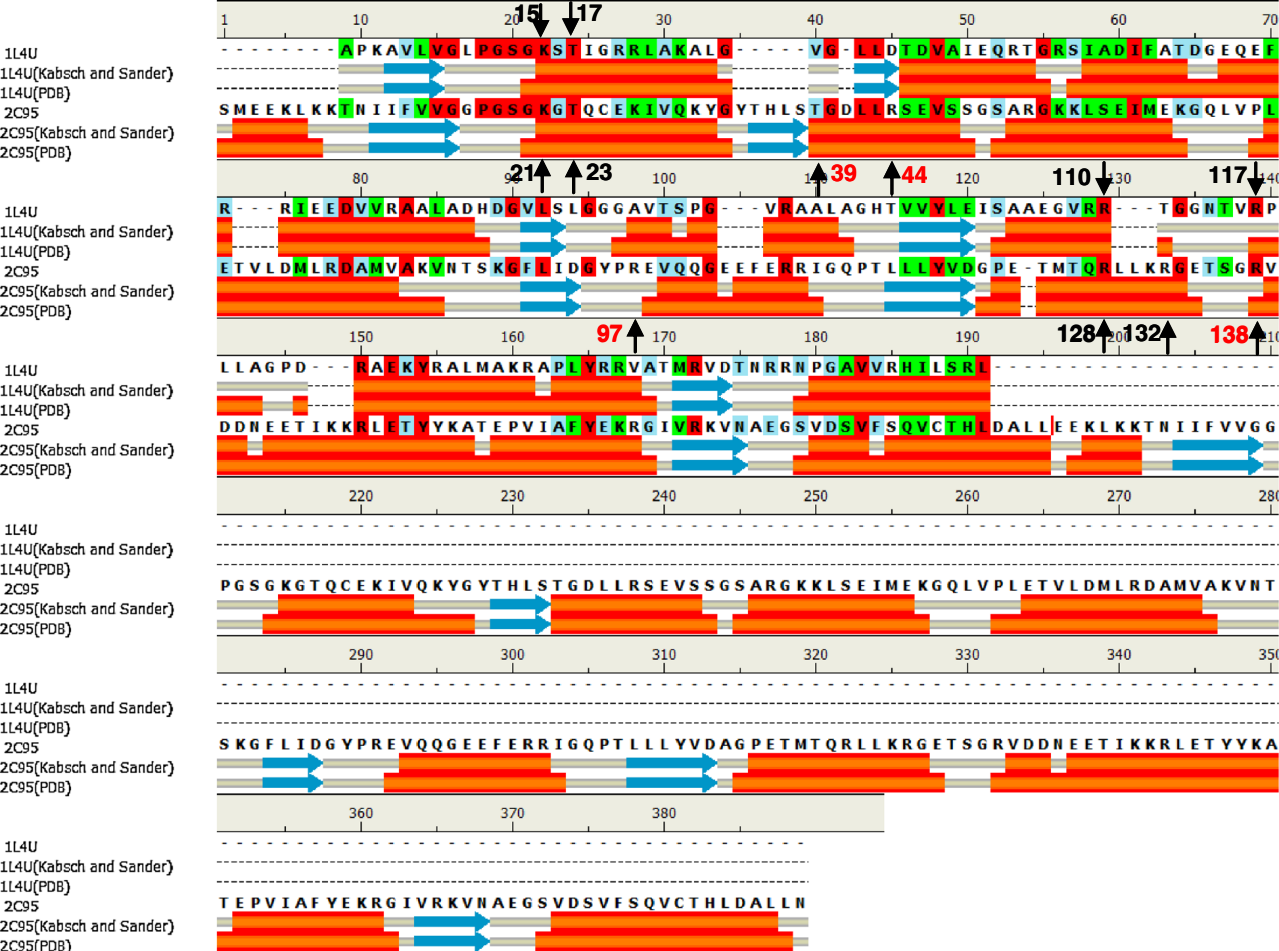
**SK and AK1 sequence alignment.** Sequence alignment of SK (pdb:1L4U) and AK1 (pdb:2C95) indicating the conserved amino acid residues making up the catalytic residues responsible for inducing the C8-H of ATP to be labile. The red numbering of the residues are from the second AK1 active site. Also indicated are the secondary structure motifs as predicted by the Discovery Studio^®^ (Accelrys Inc) assignment of secondary structure motifs according to Kabsch and Sander [25] as well as that obtained from the PDB. Back ground colours indicate; Red = identical residues, green = strong similarity, blue = weak similarity and no colour = no similarity.

The residues associated with the control and initiation of phosphoryl transfer within the active sites of SK and AK1 were identified as those close enough to ATP to enable catalysis (Table 1) [2]. The sequence alignment of SK and AK, showing the identified catalytic residues, is shown in Figure 2. While there is little, if any, sequence homology (percent identity 16.84%), it is clearly evident that the key catalytic residues associated with increasing ATP C8-H lability are conserved (Figure 2). These residues formed the basis of the SDM programme to ascertain their role in catalysis. The effect of SDM of the amino acid residues implicated in the “push” mechanism within the active sites of SK and AK1 on the specific activity of these enzymes is summarised in Table 2. In the instance of AK1, SDM was used to determine whether the mechanism involved in the second nucleotide binding site may be the putative “pull” mechanism. The mutations carried out on both SK and AK1 were: the Thr associated with the proton transfer from C8H to the α-PO_4_ (SK, Thr17; AK1, Thr 23 and Thr 39), the Arg associated with C8 protonation (SK, Arg110: AK1; Arg128 and Arg97), the Arg co-ordinated to the α-PO_4_ and β-PO_4_ (SK, Arg117; AK1, Arg132), and the Lys associated with the γ-PO_4_ protonation (SK, Lys15). The Arg and Lys mutations all significantly curtailed the specific activity of both SK and AK1, reducing their specific activity more than 100-fold. However, mutations of the initial Thr residue showed a significantly weaker effect by comparison to the Lys and Arg mutations, with the SK-T17I and the AK1-T23I mutants giving approximately 4.5 to 6 fold reduction in enzyme activity at low ATP concentrations. The inter-atomic distances between these Thr residues and the α-phosphate of ATP are 3.666 Å for SK and 4.153 Å for AK1, meaning they are in close enough proximity for direct transfer of the C8-H to the α-PO_4_ of ATP (Table 2).

**Table 1:** Catalytic residues associated with phosphoryl transfer

**Enzyme**	**SK**		**AK1A**		**AK1B**	
	**Residue**	**Interatomic distance (Å)**	**Residue**	**Interatomic distance (Å)**	**Residue**	**Interatomic distance (Å)**
1. C8-H to α-PO_4_		3.666		4.153		3.729
2. αC = O to C6-NH_2_	R153	1.893	G177	1.880	NR^1^	
3. Thr-OH to C8H	T17	3.273	T23	2.090	T39^2^	1.786
4. Thr-OH to α-PO_4_	T17	1.758	T23	2.591	T39	5.648
5. Arg-NH1 to C8	R110	4.228	R128	4.781	R97^2^	2.712
6. Arg-α-PO_4_	R117	2.757	R132	2.136	R44	1.904
7. Lys- γ-PO_4_	K15	1.882	K21	1.902	K21	1.865

NR = No coordinated residue.

Coordinated to N7.

The amino acid residues making up the “push” mechanism within the active sites of SK and AK1 identified by the inter-atomic distances between the co-crystallized nucleotide analogue and the amino acid residues within the active site. These residues included: the Thr associated with the proton transfer from C8-H to the α-PO_4_ (SK, Thr17; AK1, Thr 23), the Arg associated with C8 protonation (SK, Arg110; AK1, Arg128), the Arg co-ordinated to the α-PO_4_ and β-PO_4_ (SK, Arg117; AK1, Arg132), and the Lys associated with the γ-PO_4_ protonation (SK, Lys15; AK1, Lys21). The residues identified in the AK1 second nucleotide binding site (AK1B) are: Thr39 (associated with the proton transfer from C8-H to the α-PO_4_), Arg97 (associated with C8 protonation), Arg44/138 (co-ordinated to the α-PO_4_ and β-PO_4_), and Lys21 (associated with the γ-PO_4_ protonation).

**Table 2:** Steady state specific activities of SK and AK1, WT and mutant enzymes

**Enzyme**	**SK**		**AK1A**		**AK1B**	
	**Residue**	**Specific activity**^**1**^	**Residue**	**Specific activity**	**Residue**	**Specific activity**
1. Wild type	WT	101,89	WT	110		
2. Thr-C8H	T17I	16.73	T23V	250	T39V	19.54
3.	T17R	4.93				
4. Arg-C8H	R110A	0.92	R128A	0.128	R97A	2.61
5.			R128Q	<1	R97Q	<1
6.			R128K	<1	R97K	<1
7. Arg-α/β-PO_4_	R117	ND	R132A	0.28	R44	ND
8.			R132K	1.08		
9.			R132Q	0.00		
10. Lys- γ-PO_4_	K15I	1.665	K21	ND		
11.	K15R	0.386				

^1^ umoles/mg protein/min.

SDM of amino acid residues making up the “push” mechanism within the active sites of SK and AK1. These residues included: the Thr associated with the proton transfer from C8-H to the α-PO_4_ (SK, Thr17; AK1, Thr 23), the Arg associated with C8 protonation (SK, Arg110; AK1, Arg128), the Arg co-ordinated to the α-PO_4_ and β-PO_4_ (SK, Arg117; AK1, Arg132), and the Lys associated with the γ-PO_4_ protonation (SK, Lys15; AK1; Lys21). The residues identified in the AK1 second nucleotide binding site (AK1B) are: Thr 39 (associated with the proton transfer from C8H to the α-PO_4_), Arg 97 (associated with C8 protonation), Arg44/138 (co-ordinated to the α-PO_4_ and β-PO_4_, and Lys21 (associated with the γ-PO_4_ protonation).

### Effect of C8D-ATP on specific activity of Thr mutants

**Figure 3:**
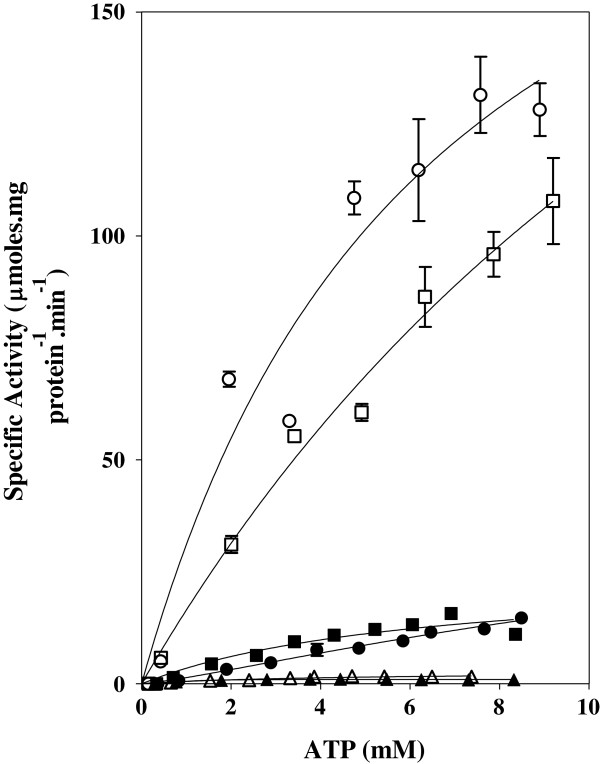
**Shikimate kinase specific activity of WT, K15I and T17I mutant enzymes.** The effect of the concentration of ATP and C8D-ATP on the specific activity and KIE of *M. tuberculosis* shikimate kinase.○ = WT SK using ATP, □ = WT SK using C8D-ATP, ● = T17I mutant using ATP, ■ = T17I mutant using C8D-ATP, Δ = K15I mutant using ATP, ▲ = K15I mutant using C8D-ATP. Final enzyme concentrations were: WT SK: 10 nM, T17I: 25 nM and K15I: 100 nM. The assays were run for 20 min (WT SK) or 80 min (T17I and K15I).

**Figure 4:**
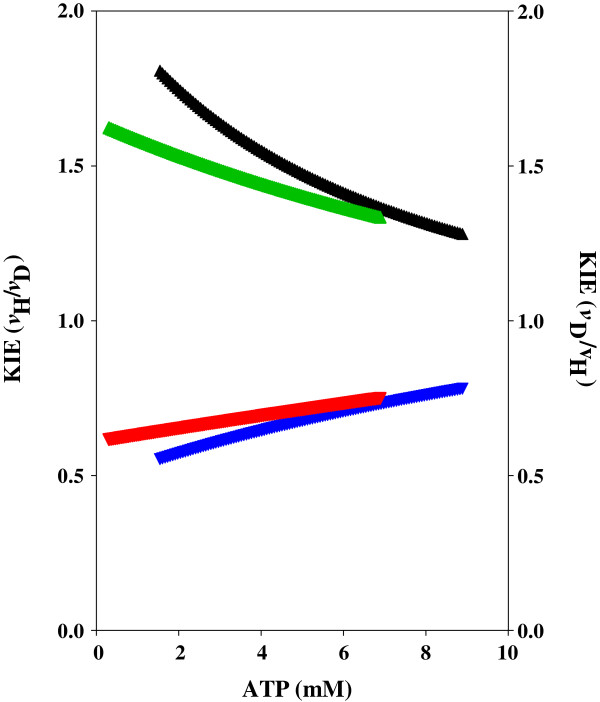
**Shikimate kinase KIE of WT and T17I mutant enzymes.** The effect of the concentration of ATP and C8D-ATP on the KIE of *M. tuberculosis* shikimate kinase. ▲ = WT KIE, (green triangle) = T17I KIE_D_, (blue inverted triangle) = WT KIE_D_, (red inverted triangle) = T17 KIE.

**Figure 5:**
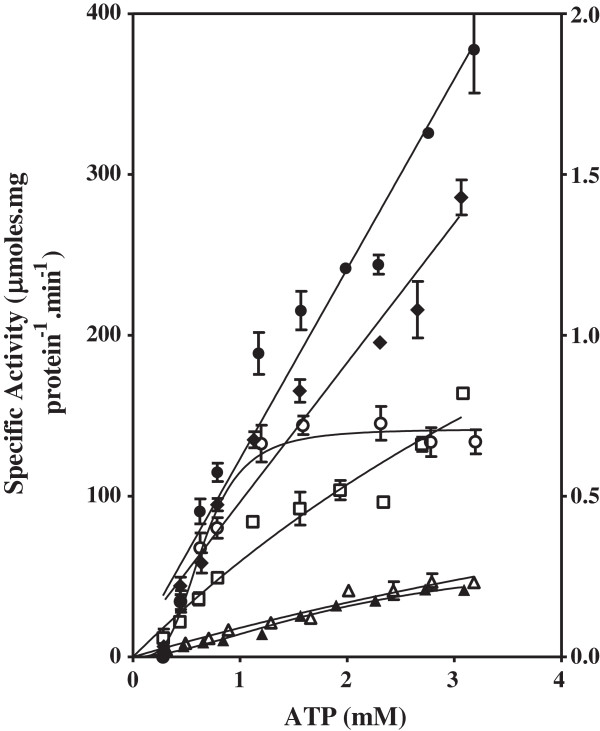
**Adenylate kinase specific activity of WT, T23V and R128A mutant enzymes.** The effect of the concentration of ATP and C8D-ATP on the specific activity of human adenylate kinase. ○ = WT AK1 using ATP, □ = WT AK1 using C8D-ATP, ● = T23V mutant using ATP, ♦ = T23V mutant using C8D-ATP, ▲ = R128A mutant using ATP, Δ = R128A mutant using C8D-ATP. R128A data on right axis. Final enzyme concentrations were: WT AK and T23V: 0.33 mM and R128A: 650 nM. The assays were run for 95 min (WT AK and T23V) or 80 min (R128A).

**Figure 6:**
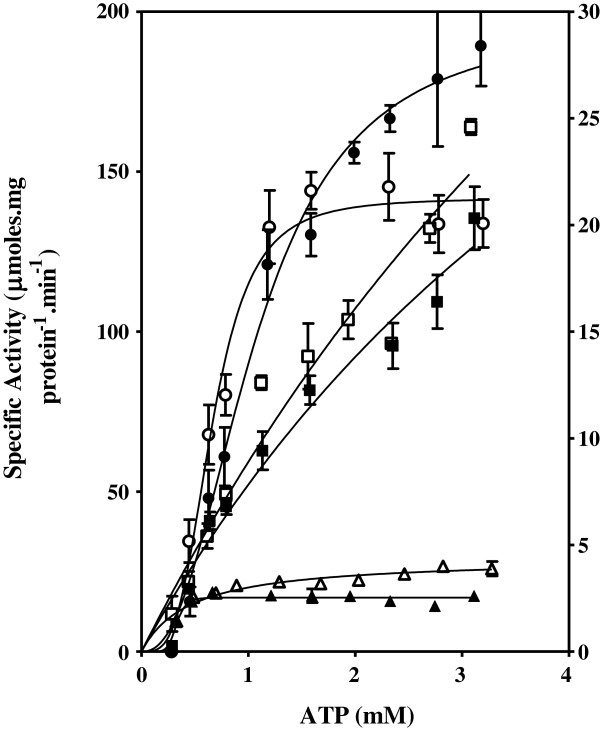
**Adenylate kinase specific activity of WT, T39V and R97A mutant enzymes.** The effect of the concentration of ATP and C8D-ATP on the specific activity of human adenylate kinase. ○ = WT AK1 using ATP, □ = WT AK1 using C8D-ATP, ● = T39V mutant using ATP, ■ = T39V mutant using C8D-ATP, ▲ = R97A mutant using ATP, Δ = R97A mutant using C8D-ATP. T39V and R97A data on right axis. Final enzyme concentrations were: WT AK: 0.33 mM, T39V: 2 nM and R97A: 20 nM. The assays were all run for 80 min.

**Figure 7:**
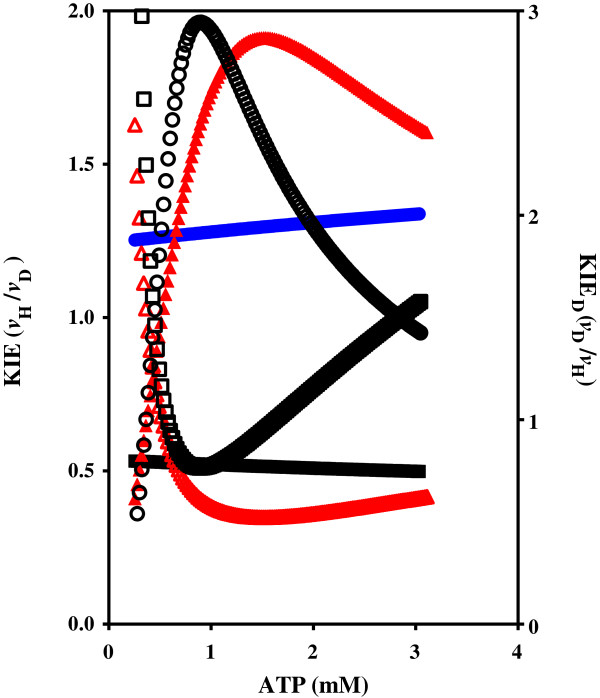
**Adenylate kinase KIE of WT, T23V, and T39V mutant enzymes.** The effect of the concentration of ATP and C8D-ATP on the KIE and KIE_D_ of human adenylate kinase. O = WT AK1 KIE, □ = WT AK1 KIE_D_, (blue circle) = AK1 T23V KIE, (black square) = AK1 T23V KIE_D_, (red triangle) = AK1 T39V KIE, (red triangle)= AK1 T39V KIE_D_.

**Figure 8:**
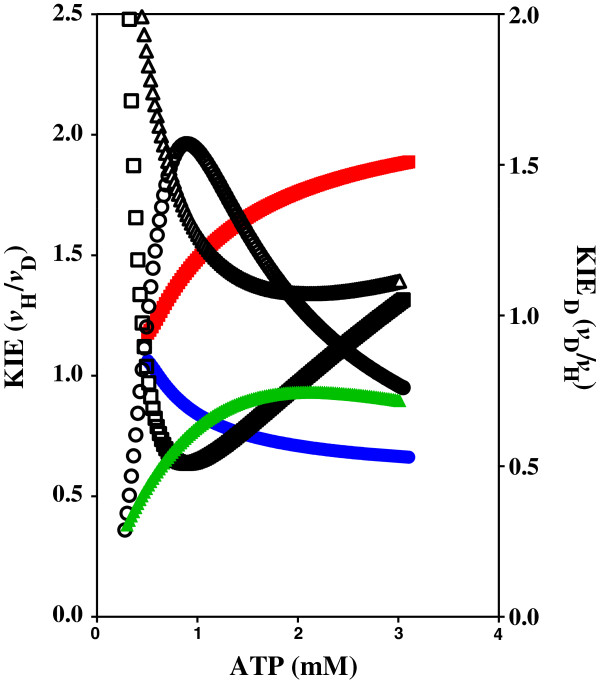
**Adenylate kinase KIE of WT, R97A, and R128A mutant enzymes.** The effect of the concentration of ATP and C8D-ATP on the KIE and KIE_D_ of human adenylate kinase. O = WT AK1 KIE, □ = WT AK1 KIE_D_, (blue circle) = AK1 R97A KIE, (red square) = AK1 R97A KIE_D_, (green triangle) = AK1 R128A KIE, Δ = R128A KIE_D_.

The effect of the ATP and C8D-ATP concentration on the steady state specific activities of wild type (WT) SK and SK-T17I, as well as WT AK1, AK1-T23V and AK1-T39V was determined (Figures 3,4,5,6,7,8). The best-fit for the data was obtained for the kinetic model using the non-linear regression algorithms within the GraphPad Prism® 5 software (Table 3). As part of the software output, a data table containing 150 data points defining the best computed fit for each enzyme’s kinetic response to the presence of either ATP or C8D-ATP. These response curves were then used to define the KIE or inverse KIE (KIE_D_) from KIE = *v*_H_/*v*_D_ or KIE_D_ = *v*_D_/*v*_H_, respectively [1]. The enzyme activity data was used to determine the values of *K*_cat_ and *K*_cat_/*K*_m_ for each enzyme (Table 3).

**Table 3:** Kinetic parameters of wild-type and mutant variants of SK and AK1

**Enzyme**	***K***_**m**_**(mM)**	***v***_**max**_	***K***_**cat**_**(s**^**-1**^**)**	***K***_**cat**_**/*K***_**m**_**(s**^**-1**^ **M**^**-1)**^
1. SK WT ATP (M-M)	6.533 6.53 ± 3.25	0.0484 233.8 ± 60.58	4.841 × 10^3^	8.749 × 10^2^
2. SK WT d-ATP (M-M)	12.74 19.26 ± 7.00	0.0503 333.4 ± 89.2	5.027 × 10^3^	3.946 × 10^2^
3. SK-T17I ATP (A-S)	Ambig 30.73 ± 2.17	Ambig 43.94 ± 3.87		
4. SK-T17I d-ATP (M-M)	22.05 ± 10.47 6.22 ± 3.57	0.0333 25.03 ± 7.70	1.331 × 10^3^	60.06
5. SK-K15I ATP (M-M)	4.054 4.13 ± 1.72	0.0057 2.736 ± 0.054	5.664 × 10	1.397 × 10
6. SK-K15I d-ATP (A-S)	1.091 0.754 ± 0.227	0.0023 0.971 ± 0.031	2.369 × 10	2.171 × 10
7. AK1 WT ATP^1^ (A-S)	1.911	0.00219	6.649 × 10^3^	3.479 × 10^3^
	0.2332	141.7		
8. AK1 WT d-ATP (M-M)	4.043	0.00250	7.583 × 10^3^	1.875 × 10^3^
	8.404	557.7		
9. AK1-T23V ATP (M-M)	12.91	0.0148	4.497 × 10^4^	3.483 × 10^3^
	12.91	1850		
10. AK1-T23V d-ATP (M-M)	10.51	0.00963	2.919 × 10^4^	2.777 × 10^3^
	.22	1062		
11. AK1-R128A ATP	Ambig	Ambig 4.597	Ambig	
	59.90			
12. AK1-R128A d-ATP	Ambig	Ambig		
	13.36	1.299		
13. AK1-T39V ATP (A-S)	5.485	0.00398	1.989 × 10^3^	3.626 × 10^2^
	1.191	29.57		
14. AK1-T39V d-ATP (M-M)	3.235	0.00159	7.972 × 10^2^	2.464 × 10^2^
	4.040	39.21		
15. AK1-R97A ATP (A-S)	0.1226	0.00131 2.532	6.527 × 10^2^	5.323 × 10^3^
	0.0002			
16. AK1-R97A d-ATP (M-M)	Ambig	0.0003 4.388	1.605 × 10^2^	
	0.4596			

The kinetic parameters of wild type SK, SK-T17I, SK-K15I, wild type AK1, AK1-T23I, AK1-T39I AK1-R97A and AK1-R128A. The datasets for each enzyme comprise values for the kinetic constants calculated using the non-linear regression analysis to calculate the *K*_cat_ (upper value), and either the best-fit data, as outlined by the GraphPad Prism5 for either the Michaelis-Menton (M-M) or Allosteric sigmoidal (A-S) fit (lower value). Ambig = Ambigous.

The SK-T17I mutant has significantly less activity than WT SK (Figure 3). WT SK showed a classical KIE, with the kinetic data reaching an asymptote at about 2 with decreasing ATP concentration, and a similar asymptote at 1 with increasing ATP concentration (Figure 4). It is interesting to note that the KIE is inverted in SK-T17I, with C8D-ATP yielding a higher specific activity than ATP (Figures 3 and 4). These inverted responses manifest in the *K*_m_ values, with the WT SK *K*_m_ for ATP and C8D-ATP being 6.533 and 12.74 mM, respectively, while the SK-T17I *K*_m_ for C8D-ATP was found to be 22.05 mM. The SK-T17I *K*_m_ could not be calculated for ATP as the response to the change in ATP concentration is linear (see discussion). The change in specificity of SK-T17I is clearly shown by the *K*_cat_/*K*_m_ values for ATP and C8D-ATP. For WT SK, these are 8.75 × 10^2^ and 3.95 × 10^2^ s^-1^.M^-1^, respectively, while the *K*_cat_/*K*_m_ for C8D-ATP for SK-T17I is 60.06 s^-1^.M^-1^.

The enzyme activity profile of the AK1-T23V mutant varies significantly from WT AK1 (Figure 5). There is no inversion of the KIE with C8D-ATP, as seen in the SK-T17I mutant. However, there is a significant increase in *v*_max_ (Figure 5, Table 3). The *v*_max_ of WT AK1 for ATP and C8D-ATP was 141.7 and 557.7 μmol.min^-1^.(mg protein)^-1^, respectively, while the *v*_max_ for ATP and C8D-ATP for AK1-T23V is 1,850 and 1,062 μmol.min^-1^.(mg protein)^-1^, respectively. These responses also manifest in the *K*_m_, with the *K*_m_ for ATP and C8D-ATP for WT-AK1 being 1.911 and 4.043 mM, respectively, while those for AK1-T23V are 12.91 and 10.51 mM, respectively. There is little change in the specificity of AK1-T23V, as shown comparing the WT AK *K*_cat_/*K*_m_ values for ATP (3.749 × 10^3^ s^-1^.M^-1^) and C8D-ATP (1.875 × 10^3^ s^-1^ M^-1^), with those for AK1-T23V (3.483 × 10^3^ s^-1^.M^-1^ for ATP and 2.777 × 10^3^ s^-1^ M^-1^ for C8D-ATP). The change in specificity within each enzyme therefore follows a classical KIE, with C8D-ATP giving a lower *K*_cat_/*K*_m_ relative to ATP.

The enzyme specific activity obtained for AK1-T39V was also significantly less than that obtained for WT AK1 (Figure 5). The *v*_max_ of AK1 for ATP and C8D-ATP was 141.7 and 557.7 μmol.min^-1^.(mg protein)^-1^, respectively, while those for AK1-T39V are 29.57 and 39.21 μmol.min^-1^.(mg protein)^-1^, respectively. These responses are reflected in the *K*_m_ values for WT AK1 in the presence of ATP and C8D-ATP being 1.911 and 4.043 mM, respectively, while those for AK1-T39V were 5.485 and 3.235 mM, respectively. The change in the specificity of AK1-T39V is shown by the *K*_cat_/*K*_m_ values for ATP and C8D-ATP, with those for WT AK being 3.479 × 10^3^ and 1.875 × 10^3^ s^-1^.M^-1^, respectively, while the *K*_cat_/*K*_m_ for ATP and C8D-ATP for AK1-T39V is 3.626 × 10^2^ and 2.464 × 10^2^ s^-1^.M^-1^, respectively. The change in specificity therefore follows a classical KIE, with C8D-ATP giving a lower *K*_cat_/*K*_m_ relative to ATP.

The KIE effect for WT AK1 is high (KIE > 2) at very low ATP concentrations, and then tends towards approximately 1 as the specific activity tends towards *v*_max_ (Figures 5 and 6). In contrast, the KIE_D_ for AK1-T23V is low at low ATP concentrations, with the KIE remaining constant over the ATP concentration range (Figures 5 and 6). The KIE and KIE_D_ of AK1-T39V is essentially the same as that obtained for WT AK1. The major effect of the T39V mutation is a significant loss in the overall enzyme activity.

### Effect of C8D-ATP on specific activity of Thr and Lys mutants

The SK-K15I mutation caused an approximately 100-fold reduction in enzyme activity (Figure 3, Table 3). The *v*_max_ of WT SK for ATP and C8D-ATP was 233.8 and 333.4 μmol.min^-1^.(mg protein)^-1^, respectively, while those for SK-K15I were 2.736 and 0.971 μmol.min^-1^.(mg protein)^-1^, respectively. The change in the specificity of the SK-K15I enzyme is shown by the *K*_cat_/*K*_m_ values for ATP and C8D-ATP for the WT SK being 8.749 × 10^2^ and 3.946 × 10^2^ s^-1^.M^-1^, respectively, while those for SK-K15I is 1.397 × 10 and 2.171 × 10 s^-1^.M^-1^, respectively. The specific enzyme activity obtained for SK-R110A was too low to allow accurate kinetic constants to be obtained.

The AK1-R97A and AK1-R128A mutations caused a significant loss in the overall enzyme activity (Figures 5 and 6). The *v*_max_ of WT AK1 for ATP and C8D-ATP was 141.7 and 557.7 μmol.min^-1^.(mg protein)^-1^, respectively, while the *v*_max_ for ATP and C8D-ATP for AK1-R97A is 2.532 and 4.388 μmol.min^-1^.(mg protein)^-1^, respectively. These responses manifest in the *K*_m_ for ATP and C8D-ATP with those for WT AK1 being 1.911 and 4.043 mM, respectively, while the *K*_m_ for ATP and C8D-ATP for AK1-R97A is 0.002 and 0.460 mM, respectively. The low activities obtained for both the AK1-R97A and The AK1-R128A enzymes did not allow for the accurate determination of the *K*_cat_, suffice to say that both mutations had a significant impact on the overall reduction of the enzyme activity. The AK1-R128A enzyme gave a linear response in enzyme activity to the change in the ATP or C8D-ATP concentration (Figure 5).

## Discussion

**Figure 9:**
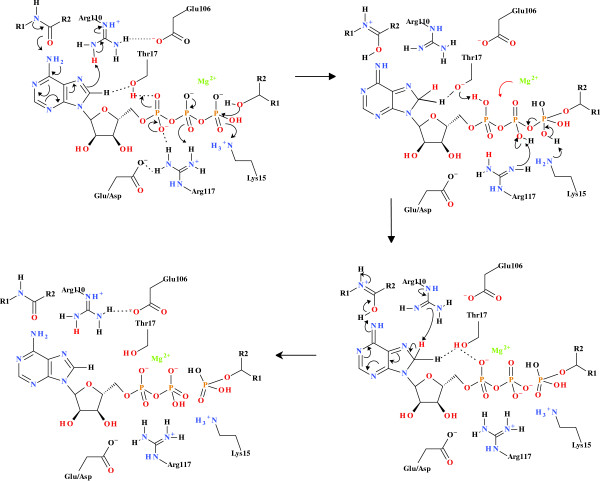
**Phosphoryl transfer mechanism found in the shikimate kinase.** Phosphoryl transfer mechanism found in shikimate kinase. The initiation of phosphoryl transfer occurs via the coordination of the ATP C6-NH_2_ to a carbonyl arising from the protein backbone by the “push” mechanism resulting in the protonation of C8 via the coordination of a conserved Arg. This renders the C8-H more acidic, allowing for the protonation of the α-PO_4_, via the conserved Thr17 carrier. There is a concomitant transfer of an H^+^ from the α-PO_4_ to β-PO_4_ via a conserved Arg, thereby facilitating the formation of the pentavalent intermediate between the γ-PO_4_ and the substrate nucleophile. There is a simultaneous ATP-mediated deprotonation of the substrate -OH, allowing for the nucleophilic attack by the substrate to create the pentavalent intermediate and allow phosphoryl transfer. A protonated Lys then transfers the proton to the γ-PO_4_, changing the Mg^2+^ from being β-PO_4_ to γ-PO_4_ coordinated to being α-PO_4_ to β-PO_4_ coordinated. The H^+^ originally arising from the C8 is then transferred back to C8, allowing the electron density of the adenyl moiety to return to the “ground-state” distribution.

**Figure 10:**
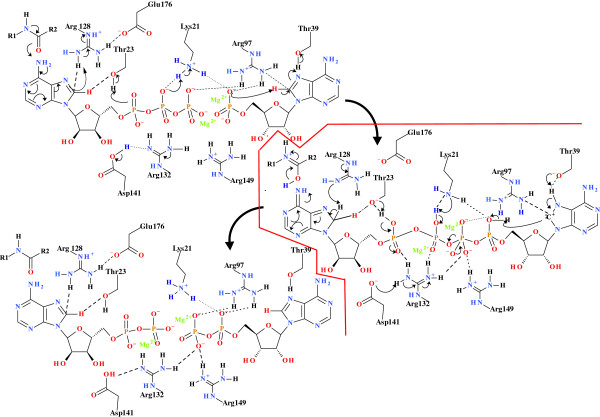
**Phosphoryl transfer mechanism found in the adenylate kinase.** Phosphoryl transfer mechanism found in adenylate kinase. The initiation of phosphoryl transfer occurs via the coordination of the AMP C6-NH_2_ to a carbonyl arising from the protein backbone by the “push” mechanism resulting in the protonation of C8 via the coordination of the conserved Arg128. This renders the C8-H more acidic, allowing for the protonation of the α-PO_4_, via the conserved Thr23 carrier. There is a concomitant transfer of an H^+^ from the α-PO_4_ to β-PO_4_ via a conserved Arg, thereby facilitating the formation of the pentavalent intermediate between the γ-PO_4_ and the substrate nucleophile. There is a simultaneous ATP-mediated deprotonation of the substrate -OH, allowing for the nucleophilic attack by the substrate to create the pentavalent intermediate and allow phosphoryl transfer. A protonated Lys then transfers the proton to the γ-PO _4_, changing the Mg^2+^ from being β-PO_4_ to γ-PO_4_ coordinated to being α-PO_4_ to β-PO_4_ coordinated. There is a concomitant binding of AMP in the second nucleotide binding site which is activated by the protonation of the N7 via the conserved Thr39 forming a carbene on the imidazole moiety causing a change in C8 hybridization from sp2 to sp3 hybridized. The carbene being stabilized by the lone pair on the coordinated Arg97. The nucleophilic attack from the oxygen on the α-PO_4_ of AMP creates the pentavalent intermediate and allows phosphoryl transfer. The H^+^ originally arising from the C8 is then transferred back to C8, allowing the electron density of the adenyl moiety to return to the “ground-state” distribution.

The “push” mechanism as defined within the Group 2 kinases (based on the kinase organization outlined by Kenyon *et al.*[2]) is found in both *Mycobacterium tuberculosis* SK and human AK1. SDM of the Arg and Lys residues in the active sites of SK and AK1 implicated in the “push” mechanism, namely, the Arg associated with C8 protonation (SK, Arg110; AK1, Arg128), the Arg co-ordinated to the α-PO_4_ and β-PO_4_ (SK, Arg117; AK1, Arg132), and the Lys associated with the γ-PO_4_ protonation (SK, Lys15; AK1; Lys21), all demonstrated that these residues are essential for catalysis in the proposed mechanism (Figures 9 and 10). AK1 has a second nucleotide binding site, and the equivalent Arg residues are those associated with C8 protonation (Arg97), and coordination to the α-PO_4_ and β-PO_4_ (Arg44/138) (Table 2). The mechanism associated with the second AK1 binding site could, however, be an example of the “pull” mechanism, as defined by the Kenyon *et al.*[2].

The threonine residues associated with the proton transfer from C8-H to the α-PO_4_ for both enzymes are Thr17 for SK, and Thr23 and Thr39 for AK1, SK-Thr17 and AK1-Thr23 appear to function as response elements towards ATP concentration in, and saturation of, the enzyme. The AK1-Thr39 has a purely hydrogen bonding role and does not appear to play a catalytic role in the creation of the pentavalent phosphorus intermediate between the ATP and the AMP. It may, however, serve to regulate the reverse reaction from 2ADP to ATP and AMP. This is shown by the varying responses to the Thr mutation, where the SK-Thr17 and the AK1-Thr23 mutations do not destroy the enzyme function but merely change the response of the enzymes to varying ATP and C8D-ATP concentrations, while the AK1-Thr39 mutation destroys functionality. The effect of deuteration of ATP on the activity and kinetic constants for the SK-T17I mutant was to invert the KIE obtained. The KIE for the WT SK enzyme was a classical (mass dependent) primary KIE (KIE ≈ 2 with decreasing ATP concentration, and asymptoting to 1 with increasing ATP concentration). The inverse KIE (KIE_D_) tends towards 0.5 at low ATP concentrations, approaching 1 with increasing ATP concentration. This trend is, however, inverted for the SK-T17I mutant enzyme where the KIE_D_ tends towards 2 with decreasing ATP concentration, while approaching 1 with increasing ATP concentration. The deuteration therefore functions in much the same manner as the Thr17 residue. The deuterium functions by increasing the lifetime of the pentavalent phosphorus species, increasing the probability for phosphorylation of the substrate. Another effect of the SK-T17I mutation is in the response of the enzyme to changing ATP concentrations. In this instance, over the concentrations used in the assays, a linear response is obtained to changing concentration is seen as opposed to a classical hyperbolic response. This demonstrates that the SK-T17I enzyme activity is based purely on an ATP concentration-dependent first order rate equation. The mechanistic role the Thr17 residue plays rests in the effective transfer of the proton from C8-H to the α-PO_4_, as part of the direct in-line mechanism of phosphoryl transfer culminating in the pentavalent phosphorus species (Figure 9). The rate of phosphoryl transfer is, therefore, based on two factors: (i) the concentration of ATP, and (ii) the induced lability of the C8-H (reflected in its apparent *pK*_a_) and subsequent proton transfer in the creation of the pentavalent transition state. At low ATP concentrations, the activity of the enzyme is predominantly governed by the release of the C8-H from ATP. As the ATP concentration increases, the role of the ATP concentration in activity begins to dominate. In the case of the WT SK enzyme, the net result of this is that the KIE is at its highest at low ATP concentrations. As the concentration effect takes over, the KIE is reduced. In the case of SK-T17I, the C8D-ATP functions more efficiently than the ATP, as the deuteration serves to increase the lifetime of the pentavalent transition state, thereby increasing the probability for the overall reaction. As the ATP concentration tends towards zero, the role of T17 in catalysis is at its maximum. As the concentration of ATP is increased, the effect of concentration on the specific activity of WT SK increases. This is borne out by the effect of ATP concentration on the activity of the SK-T17I enzyme, which shows an ATP-dependent first order response. The perceived specific activity of a particular kinase in response to varying ATP concentration is therefore dependent on the relative impacts of C8-H lability and the first order ATP concentration effect.

Examination of the data for WT AK1 indicates significant difference in the *K*_m_ values for ATP and C8D-ATP (Table 3). The *K*_m_ values for ATP and C8D-ATP for AK1-T23V were, however, similar. Deuteration significantly increases the *v*_max_ of the WT AK1 enzyme (Figure 5, Table 3). The major difference that occurs is the impact of the mutation on *v*_max_, with *v*_max_ of 141.7 for WT AK1 increasing to 1,850 for AK1-T23V in the presence of ATP, and a similar increase in *v*_max_ from 557.7 for WT AK1 to 1,062 for AK1-T23V in the presence of C8D-ATP. As also occurs in the case of SK, the mutation in the Thr residue in AK1 significantly increases the *K*_m_ of the enzyme. Another effect of the AK1-T23V mutation is in the response of the enzyme to changing ATP concentrations where, over the concentrations used in the assays, a classical hyperbolic response is not obtained and instead an almost linear response is obtained to changing concentration (Figure 5). It has been proposed that in certain kinase reactions, the protonation of the α-PO_4_ may occur directly from C8-H without the need of a carrier side-chain [2]. Here, it is proposed that for both the SK-T17I and AK1-T23V mutant enzymes, the proton transfer could occur directly from the C8-H to the α-PO_4_, albeit with reduced impact on the activity. Alternatively, the role could be fulfilled by adventitious H_2_O. The impact of H_2_O in place of the Thr hydroxy moeity would probably be concentration dependent. In the case of AK1-T23V, this may be the case, since this mutation brings about an increase in the overall activity of the enzyme. The AK1-T23V mutation does not change the specificity of the enzyme for the substrate; the influence of the Thr hydroxy group is lacking at high concentration for this mutant, however. In the case of SK-T17I, the use of C8D-ATP effectively mimics the presence of a functional T17 by stabilizing the transition state intermediate and increasing the probability of the reaction occurring, mimicking the role of the Thr hydroxy group. The loss of the interplay between the role of the Thr hydroxy group and the variation in ATP concentration due to the AK1-T23V mutation resulted in an increase in the *v*_max_. In effect, the AK1-T23V mutation merely causes this enzyme to respond in a linear first order manner to the change in the ATP concentration. The steady state enzyme activity is therefore achieved by the interplay between the two effects, namely the role of the *pK*_a_ of the C8-H and the role of the ATP concentration. In the case of the Thr mutations, H_2_O probably fulfills the role of the Thr hydroxy group and the impact of the mutation is dependent on the H_2_O concentration. In the case of SK, the availability of water to fulfill this role is lower than in the case of AK1 (as seen by the decreased overall the steady state activity of the mutant). In WT AK1, the role of Thr23 predominates at low ATP concentrations, while increasing ATP concentration steadily diminishes the importance of this role in favour of mass action. Mutant AK1-T39V enzyme appears to have lost most of its functionality (Figure 5, Table 3). The second nucleotide binding site appears to function via the “pull” mechanism, with the Thr39 serving to protonate AMP N7 - an intrinsic part of the “pull” mechanism [2]. Once this occurs, the lone pair on the nitrogen of Arg97 serves stabilize the incipient carbene which is forming at C8 of ATP (Figure 10). Removal of the Thr39 therefore destroys the functionality, even if the C8-H of the ATP is in close enough proximity of the α-PO_4_. This is because Thr39 assists this protonation event by increasing the lability of ATP C8-H in the process of stabilising the nascent carbene that forms in the “pull” mechanism. Both the R128A and the R97A mutant enzymes have significantly reduced enzyme activities, with *v*_max_ values of 2.532 and 4.597 respectively (Figure 6, Table 3). The Arg128, which functions in conjunction with Thr23, serves to protonate C8 and stabilize the carbene. Furthermore, Arg97, which functions in conjunction with Thr39, plays a key role in the formation of the carbene in the “pull” mechanism. If the C8-H of AMP was playing a direct role in the regulation of AK1 enzyme activity, a KIE should be obtained on comparison of the activity between enzyme activities obtained in the presence of AMP and C8D-AMP. This was, however, not found to be the case as AMP deuterated at the C8 position had no effect on the specific enzyme activity (Additional file 1: Figure S1).

Taking these observations into account, it should be borne in mind that the conformational changes arising on nucleotide binding within the P-loop containing nucleoside triphosphate hydrolase superfamily allow the residues of the conserved Walker A motif to “lock” the phosphate backbone into position [18,19]. As a result, two residues that form part of the consensus sequence, GXXXXGKT/S, are brought directly into play, mechanistically speaking. Significantly, these residues also play an integral part of the “push” mechanism in the guise of the conserved Thr residue coordinated to C8-H proton and the Lys residue responsible for the proton transfer between the α- and β-phosphates. The catalytic role of the conserved Thr residue has previously been identified, including its hydrogen bonding interaction via the Thr-OH^…^O-P_α_(ATP) [20]. [Shi *et al.* (1993]), in fact, ask the question, “How can Thr-23 participate in catalysis by interacting with the α-phosphate of ATP but not contribute to the energetics of catalysis?” This research demonstrates that the C8-H^…^HO-Thr and Thr-OH^…^O-P_α_(ATP) interactions play a role in the initiation of phosphoryl transfer and that the Thr residue, effectively, acts as a molecular “switch” on completion of nucleotide and substrate binding. As a result, the phosphoryl transfer occurs, via the classical in-line mechanism [21-23]. This in-line associative S_N_2 type mechanism of phosphoryl transfer is pertinent to this reaction mechanism as the phosphoryl transfer is dependent on the formation of a pentavalent phosphorus intermediate between the γ-phosphate and the substrate nucleophile.

## Conclusions

The steady-state enzyme activity in the kinase enzymes is obviously more complex than merely the phosphorylation of a substrate nucleophile. This complexity is apparent in the broad range of *K*_M_ constants of the kinases ranging from less than 0.4 μM to in excess of 1,000 μM for ATP (Carna Biosciences, Inc., Kinase Profiling Book: http://www.carnabio.com). The kinase enzymes have been classified into 25 families of homologous proteins, with the families assembled into 12 fold-groups based on the similarity of their structural folds, as well as conserved mechanisms associated with the regulation of the enzyme activity via the C8-H of the adenyl moiety [2-4]. Within a single group, both prokaryotic and eukaryotic organisms are represented with kinase isoenzymes that appear to be kinetically and functionally distinct based on the rate of phosphoryl transfer and the regulation thereof. It is therefore conceivable that the various conserved “push” and “pull” mechanisms associated with the release of C8-H, the proton transfer cascades and the resultant/concomitant creation of the pentavalent transition state are the mechanisms by which the kinase enzymes achieve this 2,500-fold variation in the *K*_M_. Polyphosphate could serve as the energy “currency” within the cell however, ATP fulfils this role. It is necessary that the kinase enzymes carry out their specific reactions at differential rates and to achieve these varying rates the kinases have evolved a number of conserved mechanisms which broadly manifest in the “push” and the “pull mechanisms”. Data outlined above serves to demonstrate the “push” mechanism associated with the role of the C8-H in the regulation of the Group 2 kinases. The phosphoryl transfer mechanism found in the Group 2 kinases (Rossmann-like fold and phosphoenolpyruvte carboxykinase-like sequences) was assessed using *M. tuberculosis* shikimate kinase and human adenylate kinase as the model systems. The initiation of phosphoryl transfer in SK occurs via the co-ordination of the ATP C6-NH_2_ to a carbonyl arising from the protein backbone by the “push” mechanism, resulting in the protonation of C8 via the co-ordination of a conserved Arg residue (Arg110). This renders the C8-H more acidic, allowing for the protonation of the α-PO_4_ via the hydroxyl of the conserved Thr carrier (Thr17). There is a concomitant transfer of an proton from the α-PO_4_ to β-PO_4_ via Arg117, thereby facilitating the formation of the pentavalent intermediate between the γ-PO_4_ and the substrate nucleophile. Where required, there is a simultaneous ATP-mediated deprotonation of the substrate -OH, allowing for the nucleophilic attack by the substrate to create the pentavalent intermediate and allowing phosphoryl transfer. In the case of adenylate kinase, the substrate is acidic, which does not require deprotonation. The protonated Lys15 then transfers the proton to the γ-PO_4_, changing the Mg^2+^ from being β-PO_4_ to γ-PO_4_ co-ordinated to being α-PO_4_ to β-PO_4_ co-ordinated. The proton originally arising from the C8 is then transferred back to C8, allowing the electron density of the adenyl moiety to return to the “ground-state” distribution. The human AK1 has two nucleotide binding sites and the second site may utilise the “pull” mechanism as outlined by Kenyon *et al.*[2]. The “pull” mechanism is the phosphoryl transfer mechanism found in the Group 4 kinases (hexokinase family with polyol substrate). The initiation of phosphoryl transfer occurs via the coordination of the ATP C6-NH_2_ to a carbonyl arising from the protein backbone by the “push” mechanism, resulting in the protonation of C8 via the co-ordination of a conserved Arg (Arg128). This renders the C8-H more acidic, allowing for the protonation of the α-PO_4_, via the hydroxyl of the conserved Thr carrier (Thr23). There is a concomitant transfer of a proton from the α-PO_4_ to the γ-PO_4_ via the conserved Arg132, thereby facilitating the formation of the pentavalent intermediate between the γ-PO_4_ and the substrate nucleophile, in this case the α-PO_4_ of the AMP in the second site. The AMP in the second nucleotide binding site is activated by the protonation of AMP-N7 via the conserved Thr39, forming a carbene on the imidazole moiety that causes a change in C8 hybridization from sp2 to sp3. The carbene is stabilized by the lone pair on the co-ordinated Arg97 residue. The nucleophilic attack from the oxygen on the α-PO_4_ of AMP creates the pentavalent intermediate and allows phosphoryl transfer. The proton originally arising from the C8 is then transferred back to C8, allowing the electron density of the adenyl moiety to return to the “ground-state” distribution. The reaction occurs via a carbene mechanism, with the carbene being stabilized via the interaction of a conserved Arg97 within hydrogen bonding distance of C8, causing C8-H to become more acidic, thereby allowing for the protonation of the α-PO_4_ via a conserved Arg97. There is a concomitant transfer of a proton from the α-PO_4_ to the γ-PO_4_ via a conserved Arg132, facilitating the formation of the pentavalent intermediate between the γ-PO_4_ and the substrate nucleophile, the α-PO_4_ of the AMP in the second site. This creates the pentavalent intermediate and allows phosphoryl transfer. The proton originally arising from the C8 is then transferred back to C8, allowing the electron density of the adenyl moiety to return to the “ground-state” distribution. The role of the C8-H of AMP in the regulation of the synthesis of ADP is unclear as deuterated AMP has no effect on the reaction (Additional file 1). This site may play a role in the regulation of synthesis of ATP and AMP from two ADP molecules in the reverse reaction using the C8-H of the ADP in the regulation.

Clearly demonstrated in the data is the perceived steady state enzyme activity is mediated via the C8-H of ATP and its transfer via a co-ordinated Thr residue. In both SK and AK1, the SDM of the Thr residue in the ATP binding site caused a loss of control of the enzyme activity as there was a significant change in the *v*_max_. This was also demonstrated by the change in the enzyme activity profile from being hyperbolic to being linear in response to the ATP concentration. As the deuteration of the C8-H has such a significant effect on the binding and steady state kinetics of kinases rationally deuterated imidazole based purine inhibitors should have improved inhibition constants when compared with their non-deuterated analogues especially at low concentrations. This has obvious implications in rational drug design.

## Methods

### Structural informatics

Discovery Studio® (Accelrys Inc) was used for all structural bioinformatics and molecular modelling protocols. The structures used were adenylate kinase (2C95.pdb) and shikimate kinase (1L4U.pdb) were obtained from the wwPDB. The AK1 structure contains bis (adenosine)-5′-tetraphosphate (AP_5_A) and the malonate ion, while the SK contains ADP, 4-(hydroxyethyl)-1-piperzine, Cl^-^ and Mg^2+^.

Unless otherwise specified, all were chemicals sourced from Merck.

### Production and site-directed mutagenesis of AK1 and SK

The *Mycobacterium tuberculosis aroK* gene, encoding the wild type SK, was obtained from the group of Chris Abell, Cambridge University, UK, in plasmid pBAN0209. A plasmid containing the gene for human AK1 (AK1A-c001) was received from the Structural Genomics Consortium (SGC) in Oxford. Both enzymes contain a N-terminal 6-His purification tag, and were transformed into *E. coli* BL21(DE3) for IPTG-induced expression. A single colony was inoculated into 50 ml LB + Amp_100_ and cultivated overnight at 37°C. 2.5 ml of this culture was transferred to 250 ml of the same medium and cultivation continued until OD_600_ ≅ 0.5-0.8. 250 μl 1 M IPTG (Peqlab Biotechnologie GmbH) was added for induction and the culture grown at 30°C overnight. The biomass from 250 ml culture was harvested, resuspended in 20 ml Binding Buffer (500 mM NaCl, 40 mM Tris pH 7.9) and sonicated for 20 min on a 50% on-off cycle. After centrifugation at 12,000 g for 10 min (4°C), the supernatant was clarified through a 0.45 μm syringe filter and loaded onto the Bio-Rad Profinia Protein Purification System fitted with a Bio-Scale Mini Profinity IMAC cartridge, using the preprogrammed native IMAC affinity-only method. The his-tagged proteins were eluted off the resin with Elution Buffer (300 mM KCl, 50 mM KH_2_PO_4_, 250 mM imidazole, pH 8.0). The purity of both enzymes was judged to be at least 90-95% by SDS-PAGE analysis (Additional file 1: Figure S1). AK1 was dialysed into Buffer A (50 mM potassium phosphate pH 7.5, 1.5 mM MgCl_2_, 120 mM KCl), while SK was dialysed into Buffer B (50 mM Tris pH 7.5, 1 M NaCl). Aliquots were snap-frozen with liquid N_2_ and stored at -75°C until required.

All mutated amino acid residues were resolved in the AK1 (2C95.pdb) and SK (1L4U.pdb) structures used. Valine was used as the amino acid of choice for the theonine mutations due to similar steric volume as threonine and as valine is hydrophobic this would limit the possibility of water replacing the threonine. Site-directed mutagenesis was performed using the Phusion Site-Directed Mutegenesis Kit (Finnzymes, Thermo Scientific) with the primer sets shown in Table 4. Each primer set also contained a silent mutation creating a restriction enzyme site, to facilitate screening. PCR reactions contained 200 μM dNTPs, 0.5 μM each primer, 3 pg/μl template DNA and 0.02U/μl Phusion DNA Polymerase. All mutations were confirmed by sequencing. The mutant enzymes were expressed and purified exactly as the wild-types.

**Table 4:** Oligonucleotide primers used for the site-directed mutagenesis of SK and AK1

**Mutant**	**5’-phosphorylated primer sets, indicating silently mutated restriction enzyme site (underlined) and new codon (bold)**	**Restriction enzyme site**
AK1-T23V	**T23V-F:** 5'-AGGGC**GTC**CAGTGTGAGAAGA-3'	*Bam*HI
	**T23V-R:** 5'-TCCCGGATCCAGGCCCA-3'	
AK1-T39V	**T39V-F:** 5'-CTACACCCACTTAAGC**GTC**GGGGAC-3'	*Afl*II
	**T39V-R:** 5'-CCATACTTCTGCACGATCTTCTCAC-3'	
AK1-R97A	**R97A-F:** 5'-ATTGATGGATATCCG**GCT**GAGGTGC-3'	*Eco*RV
	**R97-R:** 5'-CAGGAAGCCTTTGGAAGTATTGACTTTG-3'	
AK1-R97K	**R97K-F:** 5'-ATTGATGGATATCCG**AAG**GAGGTGCA-3'	*Eco*RV
	**R97-R:** 5'-CAGGAAGCCTTTGGAAGTATTGACTTTG-3'	
AK1-R97Q	**R97Q-F:** 5'-ATTGATGGATATCCG**CAG**GAGGTGC-3'	*Eco*RV
	**R97-R:** 5'-CAGGAAGCCTTTGGAAGTATTGACTTTG-3'	
AK1-R128A	**R128A-F:** 5'-ACCCAG**GCT**CTCTTGAAACGCGTAGAGAC-3'	*Mlu*I
	**R128-R:** 5'-CATGGTCTCAGGGCCTGCGT-3'	
AK1-R128K	**R128K-F:** 5'-ACCCAG**AAG**CTCTTGAAACGCGTAGAGAC-3'	*Mlu*I
	**R128-R:** 5'-CATGGTCTCAGGGCCTGCGT-3'	
AK1-R128Q	**R128Q-F:** 5'-ACCCAG**CAG**CTCTTGAAACGCGTAGAGAC-3'	*Mlu*I
	**R128-R:** 5'-CATGGTCTCAGGGCCTGCGT-3'	
AK1-R132A	**R132A-F:** 5'-ACCCAGCGGCTCCTTAAG**GCT**GGAGAGACC-3'	*Afl*II
	**R128-R:** 5'-CATGGTCTCAGGGCCTGCGT-3'	
AK1-R132K	**R132K-F:** 5'-ACCCAGCGGCTCCTTAAG**AAA**GGAGAGACC-3'	*Afl*II
	**R128-R:** 5'-CATGGTCTCAGGGCCTGCGT-3'	
AK1-R132Q	**R132Q-F:** 5'-ACCCAGCGGCTCCTTAAG**CAA**GGAGAGACC-3'	*Afl*II
	**R128-R:** 5'-CATGGTCTCAGGGCCTGCGT-3'	
SK-K15I	**K15I-F:** 5'-CTGCCGGATCCGGC**ATA**TCCACCAT-3'	*Bam*HI
	**K15-R:** 5'-GCCGACGAGAACCGCTTTGGGTG-3'	
SK-K15R	**K15R-F:** 5'-CTGCCGGGATCCGGC**AGG**TCCACCA-3'	*Bam*HI
	**K15-R**: 5'-GCCGACGAGAACCGCTTTGGGTG-3'	
SK-T17I	**T17I-F:** 5'-CTGCCGGGATCCGGCAAGTCC**ATA**ATCGGGCG-3'	*Bam*HI
	**K15-R:** 5'-GCCGACGAGAACCGCTTTGGGTG-3’	
SK-T17R	**T17R-F:** 5'-CTGCCGGGATCCGGCAAGTCC**AGA**ATCGGGCG-3'	*Bam*HI
	**K15-R:** 5'-GCCGACGAGAACCGCTTTGGGTG-3'	
SK-R110A	**R110A-F:** 5'-GGCGTGCGC**GCA**ACCGGCGGC-3'	*Eco*RV
	**R110-R:** 5'-CTCGGCGGCGGATATCTCCAGGTAG-3'	

Oligonucleotide primers used for the site-directed mutagenesis of SK and AK1, with the mutated residues in bold and the silent mutations for restriction enzyme screening underlined.

### C8D-ATP synthesis

The synthesis of ATP deuterated at the C8 position (C8D-ATP) was carried out based on the method of [1,24]. A 20 mM solution of Na_2_ATP (USB, Affymetrix) in D_2_O (Sigma) containing 60 mM triethylamine (TEA) was incubated at 60°C for 144 hours. The TEA was removed by twice passing the solution over a Dowex 20 W ion-exchange resin in the acid form. The pH of the solution was adjusted to pH 12 with NaOH prior to the second pass over the resin. The pH of the solution was adjusted to pH 6.3 prior to freeze drying. The extent of the deuteration of the C8 proton was determined by 1 H NMR and mass spectroscopy. The 1^H^ NMR was carried out on a Varian VNMRS 600 MHz NMR in D_2_O.

### AK1 assays

Steady-state specific activities were determined in assays containing 50 mM potassium phosphate buffer (pH 6.8), 0.6 mM ATP (USB, Affymetrix) and AMP (Sigma), 1 mM MgCl_2_ and differing enzyme concentrations, depending on the activity levels of the mutants. The reactions were incubated at 37°C for varying times, again depending on the activity levels of the mutants, before being terminated by the addition of 10 mM EDTA.2Na.2H_2_O.

ATP/C8D-ATP concentration gradient assays contained 50 mM potassium phosphate buffer (pH 6.8), 3.3 nM (WT AK1, AK1-T23V) or 2 nM (AK1-T39V) enzyme, and varying amounts of AMP, ATP or C8-D ATP, and MgCl_2_. These were kept at a constant ratio of 1:1:2.2 for AMP: ATP/C8-D ATP: MgCl_2_. The ATP concentrations ranged between 0.5 and 4 mM. The reactions were incubated at 37°C for varying times, before being terminated by the addition of 10 mM EDTA.2Na.2H_2_O. Each data point is the average for triplicate reactions. The ADP concentration was measured by HPLC.

### SK assays

Steady-state specific activities were determined in assays containing: 100 mM potassium phosphate buffer (pH 6.8), 500 mM KCl, 1 mM ATP and MgCl_2_, 2.4 mM shikimic acid (Sigma) and differing enzyme concentrations, depending on the activity levels of the mutants. The reactions were incubated at 37°C for varying times, again depending on the activity levels of the mutants, before being terminated by the addition of 10 mM EDTA.2Na.2H_2_O. Each data point is the average for triplicate reactions.

ATP/C8D-ATP concentration assays contained 100 mM potassium phosphate buffer (pH 6.8), 500 mM KCl, 8 mM shikimic acid, 10 nM enzyme, and varying amounts of ATP or C8D-ATP, and MgCl_2_. These were kept at a constant ratio of 1:1 for ATP/C8D-ATP: MgCl_2_. The ATP concentrations ranged between 0.5 and 10 mM. The reactions were incubated at 30°C for varying times, before being terminated by the addition of 10 mM EDTA.2Na.2H_2_O. Each data point is the average for triplicate reactions. The ADP concentration was measured by HPLC.

### HPLC analysis

The production of ADP was analysed by HPLC [1]. The assay solutions were centrifuged prior to HPLC analysis. The assays for adenosine, AMP, ADP ATP were carried out using Phenomenex 5 μ LUNA C_18_ column with the mobile phase containing PIC A® (Waters Coorporation), 250 ml acetonitrile, 7 g KH_2_PO_4_ per litre water. The flow rate of the mobile phase was 1 ml/minute with UV detection.

## Authors' contributions

CPK defined the concept and experiments of this study. RLR expressed and purified SK and AK1. RLR performed assays and collected data. CPK and RLR performed calculations on data. CPK produced C8D-ATP. Quality control by MS and NMR on C8D-ATP was carried out by CPK. CPK drafted the manuscript and RLR assisted in editing the draft manuscript. Both authors have read and approved the final manuscript.

## Supplementary Material

Additional file 1 **Figure S1.** Effect of ATP and AMP concentrations on the specific activity of AK1 showing no significant effect of deuteration of AMP on the specific activity of AK1. Assays were run in 50 mM K_2_HPO_4_/KH_2_PO_4_ buffer (pH6.8), at MgCl_2_ concentrations equal to 1.1 times the sum of the ATP and AMP concentrations. ATP and AMP were added at equivalent concentrations to the assays. ● = AMP, ■ v = deuterated AMP. Click here for file
