# Supplementary material for: The role of the C8 proton of ATP in the catalysis of shikimate kinase and adenylate kinase
Source: BMC Biochem. 2012 Aug 10;13:15. doi: 10.1186/1471-2091-13-15 (PMC3537612; doi:10.1186/1471-2091-13-15)
Supplement: Additional file 1 — Figure S1. Effect of ATP and AMP concentrations on the specific activity of AK1 showing no significant effect of deuteration of AMP on the specific activity of AK1. Assays were run in 50 mM K2HPO4/KH2PO4 buffer (pH6.8), at MgCl2 concentrations equal to 1.1 times the sum of the ATP and AMP concentrations. ATP and AMP were added at equivalent concentrations to the assays. ● = AMP, ■ v = deuterated AMP. [file 1471-2091-13-15-S1.pdf]

Additional files

Additional file 1 – Effect of deuterated AMP on the specific activity of AK1.

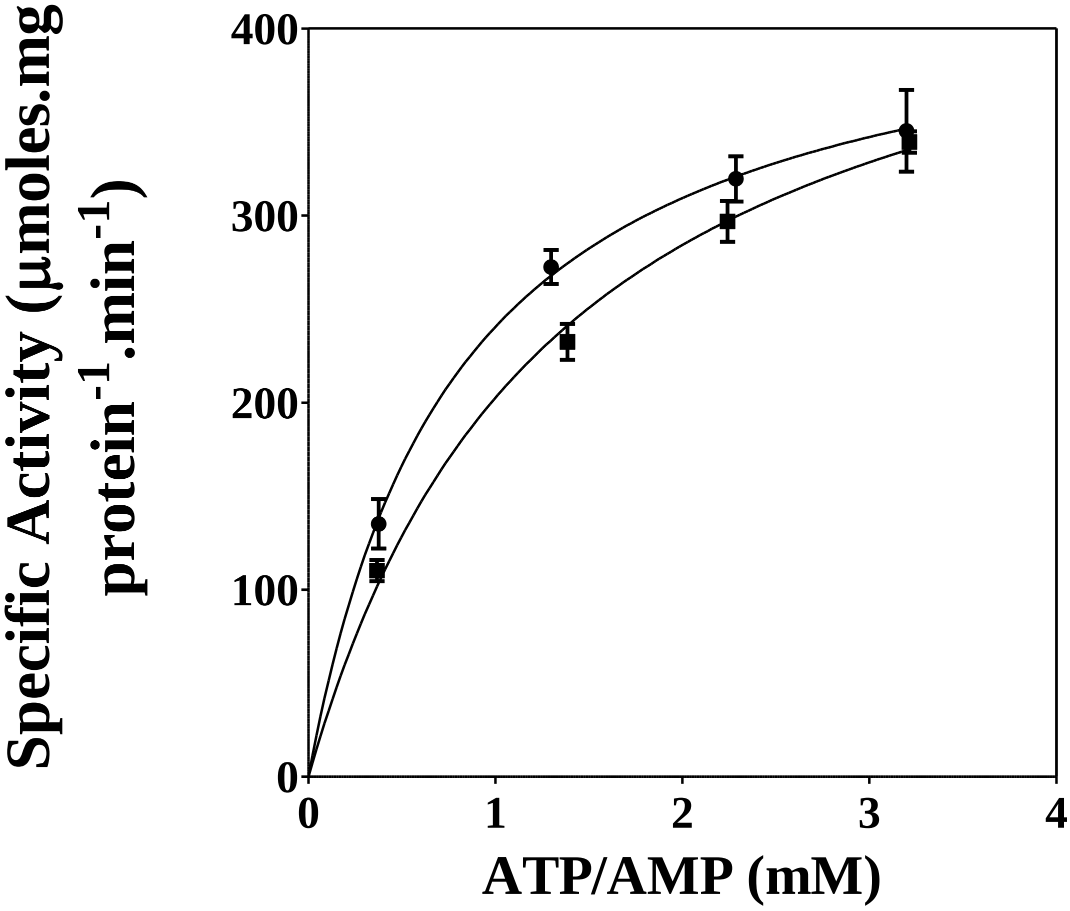

Figure 1. Effect of ATP and AMP concentrations on the specific activity of AK1 showing no significant effect of deuteration of AMP on the specific activity of AK1. Assays were run in 50 mM K_2HPO_4/ KH_2PO_4 buffer (pH6.8), at $MgCl_2$ concentrations equal to 1.1 times the sum of the ATP and AMP concentrations. ATP and AMP were added at equivalent concentrations to the assays. ● = AMP, ■ = deuterated AMP.

SDS-PAGE showing purified WT and mutated enzymes of SK and AK1.

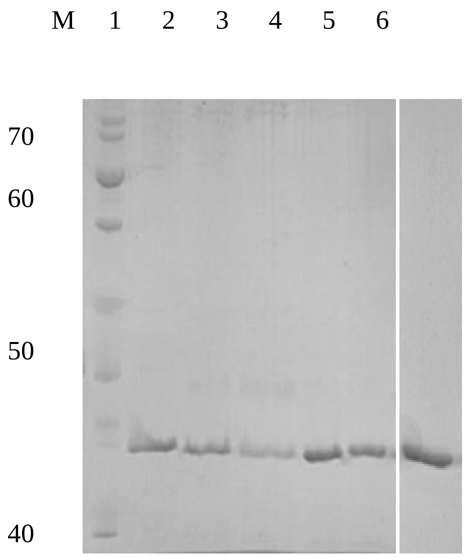

SDS-PAGE gels showing purified WT SK and various SK mutants. M = Fermentas Pageruler, with the sizes in kDa indicated on the left. 1 = K15I; 2 = K15R; 3 = T17I; 4 = T17R; 5 = R110A; 6 = WT SK.

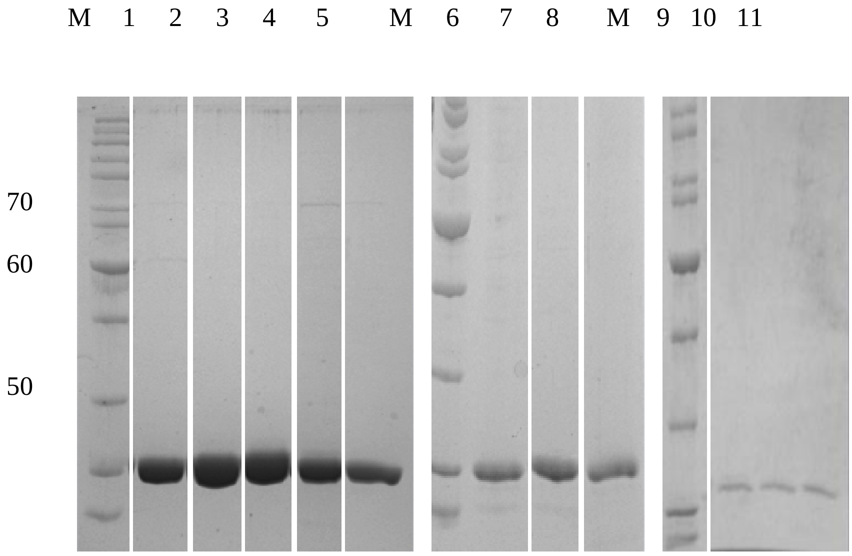

SDS-PAGE gels showing purified WT AK and various AK mutants. M = Fermentas Pageruler, with the sizes in kDa indicated on the left. 1 = WT AK; 2 = R97K; 3 = R97Q; 4 = R128K; 5 = R128Q, 6 = R132A; 7 = R132K; 8 = R132Q; 9 = R128A; 10 = R128K; 11 = R128Q

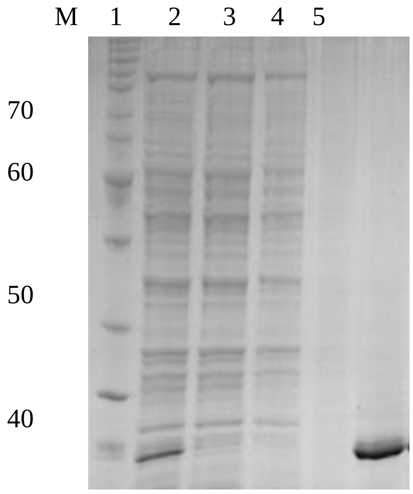

SDS-PAGE gel showing fractions taken during the purification of K15I. M = Fermentas Pageruler, with the sizes in kDa indicated on the left. 1 = crude soluble fraction, 2 = flowthrough from Profinity column, 3-4 = wash steps, 5 = eluted K15I.
